# Comparative study of exclusive breastfeeding practice among employed mothers and housewives in Tanzania

**DOI:** 10.1186/s41043-023-00407-0

**Published:** 2023-07-26

**Authors:** Oscar D. Ngao, Joanes Faustine Mboineki

**Affiliations:** Department of Nursing Management and Education, School of Nursing and Public Health, The University of Dodoma, P. O. Box 259, Dodoma, Tanzania

**Keywords:** Exclusive breast feeding, Housewives, Employed mothers, Knowledge, Sociodemographic characteristics

## Abstract

**Background:**

Exclusive breastfeeding (EBF) is a global concern as it is associated with infant protection against gastrointestinal infections, reduces newborn mortality and prevents a child from becoming overweight or obese. Even though some studies have reported high EBF practices among housewives compared to employed mothers, the influences of practices among the two groups are little documented.

**Objectives:**

The study aimed at comparing the EBF among employed mothers and housewives in Tanzania.

**Methodology:**

The study was a facility-based comparative cross-sectional study, with a sample size of 246 mothers of children less than 6 months. Convenience sampling was used to select participants. Data were collected by using a validated questionnaire and analyzed by Statistical Package for Social Science, version 23, through descriptive statistics (frequency, proportion and mean) and inferential statistics (Chi-square test (*χ*^2^) and binary logistic regression).

**Results:**

The findings show that 106 (80.9%) of housewives had good EBF compared to 78 (67.8%) employed mothers, which was a significant difference (*χ*^2^ = 5.57; *P* = 0.019). One hundred and seven (81.7%) housewives had adequate knowledge of EBF compared to 84 (73%) employed mothers, but the knowledge between them was not significantly different (*χ*^2^ = 2.63; *P* = 0.071). For employed mothers, only age was significantly associated with EBF (*χ*^2^ = 39.49; *P* = 0.006), while for housewives, none of the sociodemographic characteristics was significantly associated with EBF.

**Conclusion:**

EBF practice was higher among housewives as compared to employed mothers. Age was significantly associated with good practice of EBF among employed mothers. The effort should be strengthened to help employed mothers aged below 28 years to practice EBF. Different programs and approaches that are developed and implemented should target this age group to increase the rate of EBF.

## Introduction

Exclusive breastfeeding (EBF) is a global concern as it is associated with infant protection against gastrointestinal infections, reduces newborn mortality and prevents a child from becoming overweight or obese [1]. Globally, the rate of EBF is 43%, while in sub-Saharan Africa it IS 31% and in East Africa is 42% [2]. The trend from 2012 to 2018 shows an increment of EBF for under six children in different continents; in Africa, EBF increased by 8.6%, in America by 7.3%, Europe by 6.4% and South East Asia regions by 11.0% [3]. Furthermore, in low-income countries, EBF increased by 7.1%, in middle-income countries EBF increased by 10.9% and in upper-income countries, EBF increased by 24.1% [3].

EBF refers to when infants are not given any other foods or liquid apart from the breast milk of the mother for about the first six months after delivery [4]. Exclusive breastfeeding is important for infant because breast milk contains all nutrients required for the good growth of an infant and contains antibodies to prevent disease, protecting babies from diarrhea and acute respiratory infections [5]. Moreover, breast milk is cost-effective, reduces child malnutrition and stimulates an infant’s immune system and response to vaccination to lower the risk of infections [6].

Even though there are several reported EBF hindrances such as fear of infants becoming addicted to breast milk, lack of support from husbands, non-approval from family members, maternal or infant lack of strength due to inadequate nutrition, and lack of capacity to store human milk, unfavorable work conditions are mentioned among barriers [7]. It has been recommended by Jiawen Chen that if the workplace is near home, work schedules are flexible and workplace policies support the breastfeeding environment, the rate of EBF is likely to increase [8]. Several studies have been previously carried out to compare EBF for employed and unemployed mothers; for instance, the EBF practice in Ethiopia was 54.8% for employed mothers and 73% for unemployed [9], while in Ghana, the EBF was 16% for employed and 84% for unemployed [10], and in Somali, the EBF was 24.8% for employed and 82.9% for unemployed mothers [11]. Studies indicate that employed mothers have poor EBF practices compared to unemployed.

The number of women entering the labor force has increased and making women represent a larger workforce than ever before; however, their work has become a strong barrier for EBF [12]. With this factor, different approaches have been implemented to counteract the barrier, such as policies for maternity mandates, worksite lactation spaces, breast milk extraction breaks and supportive organizational policies [12]. The global strategy for infant and young child feeding aims to raise EBF awareness, create an environment for EBF and increase the commitment of governments, international organizations and other concerned parties to promoting EBF [13]. What has been a current change in EBF of employed mothers based on the implementation of new approaches and comparison with housewives?

In Tanzania, exclusive breastfeeding practices are improving based on demographic and health indicators, showing that for 25 years exclusive breastfeeding practices have increased from 26% in 1991–1992 to 41% in 2004–2005, to 50% in 2010 and 59% in 2015–2016 [14]. However, mothers’ occupation remains a factor determining EBF practice [15]. For instance, 50.6% of females are currently employed in Tanzania [16], with more females in human health and social work activities industry 10.8%, and more female employed in agriculture, forest and fishing industry 41.1% [17]. It is reported that the rate of employed mothers rose from 67% in 2000 to 80% in 2019, which is above the average of 63% for sub-Saharan Africa, and among the highest on the continent [18]. The more women are employed, the more effects are likely to manifest in EBF practices. Unemployed mothers in Tanzania having shown the good practices of EBF are encountering some challenges, such as mothers’ workloads and time away from infants and breast milk insufficiency [19].

For mitigating the poor practices of EBF, the government of Tanzania established three favorable laws targeting the improvement of EBF among employed mothers, for instance, (i) maternity leave (Labor Act, Art. 33): An employee is entitled to at least 84 days (12 weeks) of a paid maternity leave or 100 days if an employee gives birth to multiple infants at one time; (ii) breastfeeding leave (Labor Act, Art. 33): An employer is obligated to allow a female employee to breastfeed her infant and or child during her allotted working time, for up to a maximum of 2 h during her working days; and (iii) women’s work-while pregnant or breastfeeding (Labor Act, Art 33 & 20): Employers shall not/are not permitted to have an employee who is pregnant or nursing carry out or complete task work which is hazardous to the health of her unborn or nursing child or her health [20]. Furthermore, Tanzania has introduced three EBF policies for the population and at the hospital, including exclusive breastfeeding education, complementary food education and community health worker home visits [21]. The studies assessing changes in EBF after the introduction of EBF’s laws and policies are limited. Little is documented regarding the EBF comparison of housewives and employed mothers. Even though some studies have reported high exclusive breastfeeding practices among housewives compared to employed mothers, the influences of practices among the two groups are little documented [22]. Therefore, the study aimed at comparing the EBF among employed mothers and housewives in Tanzania. Three specific objectives are ① to compare the proportion of employed and housewife practices in EBF; ② to compare exclusive breastfeeding knowledge level among employed mothers and housewives; and ③ to determine the association of sociodemographics with exclusive breastfeeding practices among employed mothers and housewives.

## Methods

### Study area

The study was conducted in Dodoma, a region located in central Tanzania [23]. Dodoma municipal is one among seven districts in the Dodoma region; others are Chamwino, Kongwa, Mpwapwa, Bahi, Chemba and Kondoa districts [24]. The study was conducted in Dodoma because the previous data show that Dodoma region has high rates of child stunting (37.2%), low birth weight (3.7%) and underweight (17.8%) [25]. The Tanzania healthcare system is structured in hierarch in both private and public sectors, from the grassroots level of dispensaries and health centers at village and ward level, followed by district hospitals, regional referral hospitals and zonal and national hospital at highest national level [26]. In Dodoma municipal, there are 70 healthcare facilities: five hospitals (three—public and two—private), 13 health centers (eight—public and five—private) and 52 dispensaries (36—public and 16—private) [27]. Since the study was facility based, Makole health center was selected to be the study setting of this study. Makole health center is a public municipal healthcare facility catering to most of the population in the municipal, and a famous health facility for maternal healthcare services [28].

### Study design

It is a cross-sectional survey to compare the EBF among employed mothers and housewives in Tanzania.

### Study population

The study has two populations: mothers of children aged less than six months and housewives with children aged less than six months.

### Inclusion and exclusion criteria

The study included employed mothers with children aged less than six months, who attended clinics and who worked for other persons who control what is to be done and how the job is performed. Meanwhile, it included housewives with children aged less than six months who attended clinics and who stayed at home to cook, clean, take care of the children, etc., while their husbands or partners go out to work. Women were excluded from the study if they had serious sickness to impede their response, comprehension or attention in filling out the questionnaire. Moreover, they were excluded if they were unwilling to participate in the study.

### Sample size and sampling technique

The sample size was computed through G*Power version 3.1.9.4, with Chi-square statistical test. The effect size was 0.3, and the Power was 96%. The sample size of participants included in the study was 246 employed mothers and housewives. Simple random sampling was used in this study, where participants were selected randomly through a lottery method. Out of 70 healthcare facilities within Dodoma municipal, Makole health center was purposively selected because of its known optimal maternal healthcare services.

### Data collection and data collection tools

Data were collected by a researcher from May 13 to June 10, 2022, through a structured self-administered questionnaire. For women who were unable to read or write, the interviewer-administered questionnaire was used. The questionnaire was adopted from Cheko [2]. Later, it was modified to match the study objectives. Through a pilot test finding, the questionnaire was validated and the final version had three parts: (1) sociodemographic data of participants, (2) exclusive breastfeeding practices and (3) knowledge of exclusive breastfeeding practice.

### Data analysis plan

Data were entered and analyzed in Statistical Package for Social Science (SPSS), version 23. Descriptive analysis was performed for the sociodemographic characteristics (age, occupation, education, religion, gravidity and others). Categorical variables were analyzed through frequencies and percentages, while continuous data were analyzed using means. Chi-square was used to determine the association between sociodemographic characteristics and exclusive breastfeeding practice. The binary logistic regression was performed to determine the extent to which sociodemographic factors were associated with the EBF. Data were considered significant, only if the *P* value was less than 0.05.

### Variable definitions

Employed mothers are women who worked for other persons who control what is to be done and how the job is performed, while housewives are women who stayed at home to cook, clean, take care of the children, etc., but their husbands or partners go out to work.

### Variables and variables measurements

This study has independent and dependent variables, whereby maternal data (gravidity, type of delivery and place of delivery) and sociodemographic characteristics (age, marital status. occupation and education level) are categorized into the independent variables. The dependent variable was EBF practice and knowledge among mothers with children less than 6 months old. For measurements of sociodemographic data, the categorical variables were summed in the form of frequencies and percentages, while the continuous variables were in means and standard deviation. For measurement of EBF practices, six questions were used to assess the EBF practices, whereby every correct answer was given a score of one, and incorrect answers were given a zero score. Mothers who scored three or more of the total questions were considered to have a good practice of EBF, and those who scored below three points were considered to have a poor practice of EBF. For measurement of the knowledge level of EBF, 12 questions were used to determine the knowledge level. The correct answer per each question was awarded a score of one, and the incorrect was given a zero score. Mothers who scored six points and above were considered to have adequate knowledge about EBF, and those who scored below six points were considered to have inadequate knowledge.

### Ethical consideration

The ethical clearance has obtained from the University of Dodoma Institution Research Review Committee (UDOM-IRREC). The committee revealed that the study had no potential harm to respondents. Informed consent was obtained from each respondent before the commencement of the study. The confidentiality of participants' data was maintained by restricting participants from disclosing their actual names; rather, codes were used. Participants were free to participate or withdraw from the study anytime they felt to do so.

## Results

### Sociodemographic characteristics of respondents

In this study, 115 (46.7%) were employed mothers and 131 (53.3%) were housewives. The average age of both housewives and employed mothers was 28 (18–41) years, with a standard deviation of 5.7. Most of the participants 123 (50%) had a secondary education level. Moreover, for both employed mothers and housewives, the majority were married 139 (56.5%), Christian 136 (55.3%), multipara 163 (66.3%), delivered at the health facility 171 (69.5%) and delivered by spontaneous vaginal 196 (79.7%) (refer to Table 1). In comparing the sociodemographic of employed mothers and housewives, most employed mothers (53.9%) had secondary school education than their counterparts (*χ*^2^ = 39.64; *P* < 0.001). The majority of housewives (65.6%) were married compared to 46.1% of employed mothers (χ^2^ = 20.26; *P* < 0.001). Meanwhile, most housewives (85.5%) had undergone normal spontaneous vaginal delivery (SVD) compared to 73% of employed mothers (χ^2^ = 5.86; *P* = 0.18) (refer to Table 2).

**Table 1:** Sociodemographic characteristics of both housewives and employed mothers (*n* = 246)

Variable	Frequency (*n*)	Percentage (%)
Age		
Mean (SD) 28 ± 5.7 Range (18–41)		
Occupation		
Employed	115	46.7
Housewives	131	53.3
Education level		
College level and above	70	28.5
Secondary level	123	50.0
Primary level	44	17.9
Never gone to school	9	3.7
Marital status		
Single	87	35.4
Married	139	56.5
Divorced/widow	20	8.1
Religion		
Christian	136	55.3
Muslim	110	44.7
Delivery place		
Health facility	171	69.5
Home	75	30.5
Delivery type		
Spontaneous vaginal delivery (SVD)	196	79.7
Caesarian Section (C/S)	50	20.3
Gravidity		
Prime Para	83	33.7
Multipara	163	66.3

SD standard deviation

**Table 2:** Comparison of sociodemographic characteristics of housewives and employed mothers

Sociodemographic characteristics	Employed women *n* (%)	Housewives *n* (%)	*χ*^2^ (*P *value)	Confidence interval	
				Lower	Upper
Age mean (SD) 28 ± 5.7 Range (18–41)			32.197 (0.056)	0.036	0.046
Education level					
College level and above	47 (40.9)	23 (17.6)	39.64 (< 0.001) *	0.0	0.0
Secondary level	62 (53.9)	61 (46.6)			
Primary level	6 (5.2)	38 (29)			
Never attended school	–	9 (6.9)			
Marital status			20.26 (< 0.001) *	0.0	0.0
Married	53 (46.1)	86 (65.6)			
Single	57 (49.6)	30 (22.9)			
Divorced/widow	5 (4.3)	15 (11.5%)			
Religion			0.022 (0.898)	–	–
Christian	63 (54.8)	73 (55.7)			
Muslim	52 (45.2)	58 (44.3)			
Delivery place			0.00 (1.00)	–	–
Health facility	80 (69.6)	91 (69.5)			
Home	35 (30.4)	40 (30.5)			
Delivery type			5.86 (0.18) *	–	–
Normal (SVD)	84 (73)	112 (85.5)			
C/Section	31 (27)	19 (14.5)			
Gravidity			1.29 (0.281)	–	–
Prime para	43 (37.4)	40 (30.5)			
Multipara	72 (62.6)	91 (69.5)			

^*^A significant difference *P*-value

### EBF practices among employed mothers and housewives

In item analysis, most housewives 92 (70.2%) initiated EBF within 1 h after delivery, 107 (81.7%) gave their babies colostrum and 105 (80.2%) breastfed babies more than eight times per day. For employed mothers, 69 (60.0%) initiated EBF within 1 h after delivery, 73 (63.5%) gave their babies colostrum and 90 (78.3%) exclusively breastfed the baby for 6 months and above. Generally, most of the housewives 106 (80.9%) practice exclusive breastfeeding practice than employed mothers 78 (67.8%) (refer to Table 3). Through a cross-tabulation, the Chi-square test (χ^2^) was performed to compare the EBF practices between housewives and employed mothers. The findings show that 106 (80.9%) of housewives had good EBF compared to 78 (67.8%) employed mothers, which indicates a significant difference in EBF between employed mothers and housewives (*χ*^2^ = 5.57; *P* = 0.019) (refer to Table 4).

**Table 3:** EBF practices among employed mothers and housewives

Variables	Employed *n* (%)	Housewives *n* (%)
Initiating EBF within 1 h after delivery		
Yes	69 (60.0)	92 (70.2)
No	46 (40.0)	39 (29.8)
Breastfeed baby exclusively		
Yes	79 (68.7)	104 (79.4)
No	36 (31.3)	27 (20.6)
Giving colostrum/ first milk to baby		
Yes	73 (63.5)	107 (81.7)
No	42 (36.5)	24 (18.3)
Breastfeed baby more than 8 times per day		
Yes	82 (71.3)	105 (80.2)
No	33 (28.7)	26 (19.8)
Giving baby anything before initiating breastfeed		
Yes	93 (80.9)	103 (78.6)
No	22 (19.1)	28 (21.4)
Exclusive breastfeeding practice		
Yes	78 (67.8)	106 (80.9)
No	37 (32.2)	25 (19.1)

**Table 4:** EBF practices among employed mothers and housewives

Category	Good practice of EBF *n* (%)	Poor practice of EBF *n* (%)	*χ*^2^ (*P *value)
Occupation			5.57 (0.019)
Employed	78 (67.8)	37 (32.2)	
Housewives	106 (80.9)	25 (19.1)	

### Comparing EBF knowledge level between employed mothers and housewives

In item analysis, most of the housewives, 98 (74.8%) had heard about EBF, 101 (77.1%) knew the EBF importance, 101 (77.1%) understood that breast milk alone can sustain a baby for 6 months, 100 (76.35%) knew that a baby should be exclusively breastfed and 97 (74.0%) knew that semi-solid food should be introduced at 6 months. For employed mothers, 76 (66.1%) heard about EBF, 74 (64.3%) knew the EBF importance, 76 (66.1%) knew that breast milk only can sustain a baby for 6 months and 76 (66.1%) knew semi-solid food should be introduced at 6 months (refer to Table 5). Through a cross-tabulation, 107 (81.7%) housewives had adequate knowledge of EBF compared to 84 (73%) employed mothers, but the knowledge between them was not significantly different (*χ*^2^ = 2.63; *P* = 0.071) (refer to Table 6).

**Table 5:** Knowledge on exclusive breastfeeding among employed mothers and housewives

Variables	Employed *n* (%)	Housewives *n* (%)
Meaning of EBF		
Yes	103 (89.6)	102 (77.9)
No	12 (10.4)	29 (22.1)
Importance of EBF		
Yes	74 (64.3)	101 (77.1)
No	41 (35.7)	30 (22.9)
Heard about EBF		
Yes	76 (66.1)	98 (74.8)
No	39 (33.9)	33 (25.2)
Breast milk alone is enough for infant < 6 months		
Yes	105 (91.3)	110 (84.0)
No	10 (8.7)	21 (16.0)
Breast milk alone can sustain baby for 6 months		
Yes	76 (66.1)	101 (77.1)
No	39 (33.9)	30 (22.9)
EBF protects mother from pregnancy		
Yes	77 (67.0)	100 (76.3)
No	38 (33.0)	31 (23.7)
EBF protects baby from illness		
Yes	104 (90.4)	102 (77.9)
No	11 (9.6)	29 (22.1)
Expressed breast milk should be feed to the baby		
Yes	74 (64.3)	100 (76.3)
No	41 (35.7)	31 (23.7)
Semi-solid food to be introduced at 6 months		
Yes	76 (66.1)	97 (74.0)
No	39 (33.9)	34 (26.0)
Is it important to give a newborn child other foods such as porridge, tea and juice?		
Yes	76 (66.1)	89 (67.9)
No	39 (33.9)	42 (32.1)
Frequent sucking helps for milk production		
Yes	76 (66.1)	100 (76.3)
No	39 (33.9)	31 (23.7)
Pre-lacteal feeding needed for an infant before starting breast milk		
Yes	106 (92.2)	121 (92.4)
No	9 (7.8)	10 (7.6)
Knowledge on exclusive breastfeeding		
Adequate knowledge	84 (73.0)	107 (81.7)
Inadequate knowledge	31 (27.0)	24 (18.3)

**Table 6:** Comparing knowledge level of EBF between employed mothers and housewives

Category	Adequate knowledge of EBF *n* (%)	Inadequate knowledge of EBF *n* (%)	*χ*^2^ (*P *value)
Occupation			2.63 (0.071)
Employed	84 (73)	31 (27)	
Housewives	107 (81.7)	24 (18.3)	

### Association of sociodemographic characteristics with EBF among employed mothers and housewives

For employed mothers, age was significantly associated with EBF (*χ*^2^ = 39.49; *P* = 0.006), while other sociodemographic characteristics were not associated with EBF. For housewives, none of the sociodemographic characteristics was significantly associated with EBF (refer to Table 7). The binary logistic regression was performed to determine the extent to which the age of employed mothers was associated with EBF. It was found that employed mothers aged 28 years were 2.1 (OR) times more likely to practice EBF, *P* < 0.001. Meanwhile, in comparing sociodemographic characteristics between groups (employed mothers and housewives), it shows that there were significant age differences (*χ*^2^ = 4.902; *P* < 0.001), occupation (*χ*^2^ = 5.566; *P* = 0.018) and gravidity (*χ*^2^ = 4.837; *P* = 0.028) (refer to Table 8).

**Table 7:** Association of sociodemographics with EBF practices among employed mothers and housewives

Sociodemographic characteristics	Housewives					Employed mothers				
	EBF good practice n (%)	EBF poor practice n (%)	χ^2^ (*P-*value)	Confidence interval		EBF good practice *n* (%)	EBF poor practice *n* (%)	*χ*^2^ (*P *value)	Confidence interval	
			Low. Upp	Low.	Upp.				Low.	Upp.
Age mean = 28 (18–40)			30.86 (0.057)	0.043	0.054	–	–	39.49 (0.006) *	0.000	0.002
Highest educational level			2.12 (0.547)	0.581	0.606			1.45 (0.483)	0.459	0.485
College level and above	18 (17)	5 (20)				29 (37.2)	18 (48.6)			
Secondary level	52 (49.1)	9 (36)				45 (57.7)	17 (45.9)			
Primary level	30 (28.3)	8 (32)				4 (5.1)	2 (5.4)			
Never gone to school	6 (5.7)	3 (12)				–	–			
Marital status			1.35 (0.51)	0.540	0.566			1.27 (0.529)	0.560	0.585
Married	72 (67.9)	14 (56)				38 (48.7)	15 (40.5)			
Divorced/Widow	11 (10.4)	4 (16)				4 (5.1)	1 (2.7)			
Single	23 (21.7)	7 (28)				36 (46.2)	21 (56.8)			
Religion			3.32 (0.069)	–	–			0.01 (0.914)	–	–
Christian	55 (51.9)	18 (72)				43 (55.1)	20 (54.1)			
Muslim	51 (48.1)	7 (28)				35 (44.9)	17 (45.9)			
Place of delivery			0.09 (0.76)	–	–			0.013 (0.91)	–	–
Health facility	73 (68.9)	18 (72)				54 (69.2)	26 (70.3)			
Home	33 (31.1)	7 (28)				24 (30.8)	11 (29.7)			
Type of delivery			0.16 (0.693)	–	–			1.85 (0.173)	–	–
Normal	90 (84.9)	22 (88)				60 (76.9)	24 (64.9)			
Caesarian section	16 (15.1)	3 (12)				18 (23.1)	13 (35.1)			
Gravidity			1.31 (0.253)	–	–			2.95 (0.086)	–	–
Multipara	76 (71.7)	15 (60)				53 (67.9)	19 (51.4)			
Prime para	30 (28.3)	10 (40)				25 (32.1)	18 (48.6)			

^*^Represent a significant difference *P* value

**Table 8:** Factors associated with EBF practice among employed mothers and housewives

Variables	Exclusive breastfeeding		χ^2^	*P* value
	Yes *n* (%)	No *n* (%)		
Age group (years)				
18–25	75 (70.0)	37 (33.0)	4.902	< 0.001
26–33	65 (76.5)	20 (23.5)		
34–41	44 (89.8)	5 (10.2)		
Occupation				
Employed	78 (67.8)	37 (32.2)	5.566	0.018
Housewives	106 (80.9)	25 (19.1)		
Educational level				
No formal education	6 (66.6)	3 (33.3)		
Primary level	34 (77.3)	10 (22.7)	3.712	0.294
Secondary level	97 (78.9)	26 (21.1)		
College	47 (67.1)	23 (32.9)		
Marital status				
Single	59 (67.8)	28 (32.2)	3.638	0.162
Married	110 (79.1)	29 (20.9)		
Divorced/widow	15 (75.0)	5 (25.0)		
Religion				
Christian	98 (72.1)	38 (27.9)	1.209	0.271
Muslim	86 (78.2)	24 (21.8)		
Delivery place				
Health facility	127 (74.3)	44 (25.7)	0.083	0.773
Home	57 (76.0)	18 (24.0)		
Delivery type				
Normal (SVD)	150 (76.5)	46 (23.5)	1.538	0.215
C/Section	34 (68.0)	16 (32.0)		
Gravidity				
Prime para	55 (66.3)	28 (33.7)	4.837	0.028
Multipara	129 (79.1)	34 (20.9)		

## Discussion

### The proportion of employed and housewives who practice exclusive breastfeeding

The study shows a high proportion of housewives 80.9% practicing EBF than employed mothers 67.8%. The findings are almost consistent with the previous study's reported EBF of 73% for unemployed mothers and 54.8% for employed mothers [9]. Furthermore, a high proportion of EBF was observed among non-working women (75.5%) compared to working women 26.5% [29]. Therefore, the study finding from the current study conforms to the previous findings that housewives or non-employed mothers have a high proportion of EBF practices than their counterparts.

### Comparison of the knowledge level of EBF among employed mothers and housewives

The study shows a high proportion of 81.7% of housewives with adequate knowledge of EBF than employed mothers 73%, but not significantly different (*P* = 0.071). The reason for the non-significant difference might be since both women regardless of their occupation receive similar knowledge when visiting reproductive child health for maternal visits. The findings are consistent with the study carried out in Ethiopia, showing high proportion (81.4%) of housewives had knowledge about EBF than employed mothers (80.1%), without a statistical difference [2].

### Association of sociodemographics with EBF practices among employed mothers and housewives

It was found that the age of employed mothers was associated with EBF, especially those aged 28 years. This could be the reason that women 28 years old are adults who have good cognitive ability, especially they abstractly think about the importance of EBF. Moreover, women aged 28 years might have positive health beliefs about EBF, especially believing in risk susceptibility to their children that lead to the practice of EBF.

### Study limitation

A purposive selection of Makole health center as a study setting out of 69 healthcare facilities might have affected the findings. Moreover, the use of non-probability sampling of participants especially convenience sampling might have affected the statistical test results. The study assessed fewer sociodemographic characteristics and maternal factors, which indicates that some of the variables left unstudied could have a significant influence on EBF.

## Conclusion

EBF practice was higher among housewives as compared to employed mothers. Age was significantly associated with good practice of EBF among employed mothers. The effort should be strengthened to help employed mothers aged below 28 years to practice EBF. Different programs and approaches that are developed and implemented should target this age group to increase the rate of EBF.

## Data Availability

The datasets of the current study are available from the corresponding author on reasonable request. Point of contact: Dr. Joanes Faustine Mboineki, email: 624639045@qq.com or joanesmboineki@gmail.com, mobile number: + 255756310634.
